# Relationship between greenhouse gas emission, energy consumption, and economic growth: evidence from some selected oil-producing African countries

**DOI:** 10.1007/s11356-020-08065-z

**Published:** 2020-02-22

**Authors:** Abdulmalik M. Yusuf, Attahir Babaji Abubakar, Suleiman O. Mamman

**Affiliations:** 1 WASCAL, Climate Change Economics, Cheikh Anta Diop University, Dakar, Senegal; 2 Department of Economics, Ahmadu Bello University, Zaria, Nigeria; 3 Department of Economics, Business School, University of Aberdeen, Aberdeen, United Kingdom

**Keywords:** Greenhouse gas emission, Energy consumption, Economic growth, Oil, Environmental Kuznets curve (EKC), Environment degradation, Africa

## Abstract

This paper investigates the relationship between greenhouse gas emissions, energy consumption, and output growth among African OPEC countries (Libya, Nigeria, Angola, Algeria, Equatorial Guinea, and Gabon) using the panel autoregressive distributed lag model (PARDL) estimated by means of mean group (MG) and pooled mean group (PMG) for the period 1970–2016. The paper estimated three panel models comprising the components of greenhouse gasses which includes nitrous oxide, carbon dioxide (CO2), and methane and examined their relationship with economic growth and energy consumption. The findings of the study showed evidence of a positive impact of economic growth on both CO2 and methane emissions in the long run. Its impact on nitrous oxide emissions although positive was found to be statistically insignificant. Energy consumption was also found to produce an insignificant positive impact on CO2, methane, and nitrous oxide emissions in the long run. In the short run, economic growth exerts a significant positive effect on methane emissions; however, its effect on CO2 and nitrous oxide emissions although positive was found to be statistically insignificant. Energy consumption produces an insignificant impact on all components of greenhouse gasses in the short run. In addition, our empirical results showed the presence of a non-linear relationship between methane emissions and economic growth, confirming the existence of the environmental Kuznets curve (EKC) only in the case of methane emissions model.

## Introduction

Environmental degradation continues to be a serious challenge in Africa, particularly in oil-producing African countries. This largely due to the hazards associated with oil extraction and refining activities. These activities entail the exhaustion of carbon which thus produces a negative effect on the environment via greenhouse gasses emission. Arguments in extant literature posit that the growth of economic activities and energy consumption is associated with increasing greenhouse gas emission, largely due to utilization of non-efficient energy methods (see Saidi and Hammami, 2015; Muhammad 2019). An increase in greenhouse gasses emission portends danger for the environment and humanity via its negative implication on climate change.

Discussions about climate change in recent times are being focused on human-induced factors that contribute to climate change, this is even though both human and natural factors contribute to climate change. The reason for the focus on human-led factors is because they can largely be avoidable (Schellnhuber et al. 2016; IPCC 2014). As noted by Stern (2006), the negative effect of climate change on countries is not on equal footing, poor nations, and individuals are likely to suffer the negative consequences earlier and the most; hence, the need for concerted efforts to mitigate the challenge of climate change before it exacerbates further.

Greenhouse gasses such as nitrous oxide (N_2_O), carbon dioxide (CO_2_), and methane are regarded as important contributors to climate change while at the same time seen as products of economic activities that drive economic growth and development (Mladenović et al. 2016). If efforts to combat climate change are inadequate, climate change has the tendency of negatively impacting development strides and economic growth efforts of countries. Despite the effect of climate change is global, oil-producing African countries are expected to be gravely affected by it. This is predicated upon the fact that crude oil exploration techniques which lead to oil spillage and environmental degradation are largely adopted; further, the high volume of gas flaring and ineffective implementation of environmental laws contributes to further environmental degradation. On the economic effect of climate change, Amjath-Babu et al. (2016) noted that climate change has the tendency to contribute towards the deterioration of both human and social development potentials.

Extant literature on economic activity and environmental nexus indicates the existence of two opposing schools of thought which can be termed the optimistic and pessimistic schools (Alagidede et al. 2015). The pessimistic school is of the view that ensuring the sustainability of the environment requires the need to suspend economic growth; this is because to attain economic growth, energy, and raw materials are sourced from the environment, and also, economic waste is dumped back to the environment. Contrary to this view, the optimistic school noted that the fears alluded to by the pessimistic school can be mitigated via technological change and application of techniques that limit the harmful effects on the environment such as green technologies. As a result, both environmental sustainability and economic advancement can be achieved simultaneously (Alagidede et al. 2015).

Unlike existing studies such as Apergis and Payne 2009; Apergis and Payne 2010; Narayan and Narayan 2010; Richmond and Kaufmann 2006; Lean and Smyth 2010 which relied on the use of a single measure of greenhouse gas emission (carbon dioxide), this study extend on existing literature by incorporating other components of greenhouse gas emission namely methane (NH_4_) and nitrous oxide (N_2_O). In addition, the study examined whether the EKC holds for all components of greenhouse gasses by extending it to methane and nitrous oxide emissions; this is an extension to existing studies that relied on CO2 emissions alone to verify the hypothesis. On the methodological front, the study employed the mean group (MG) and pooled mean group (PMG) being components of panel ARDL which deals with non-stationary of series in a heterogeneous panel and account for endogeneity in examining both long-run and short-run relationship between study variables.

## Literature review

### Theoretical consideration

**Fig. 1:**
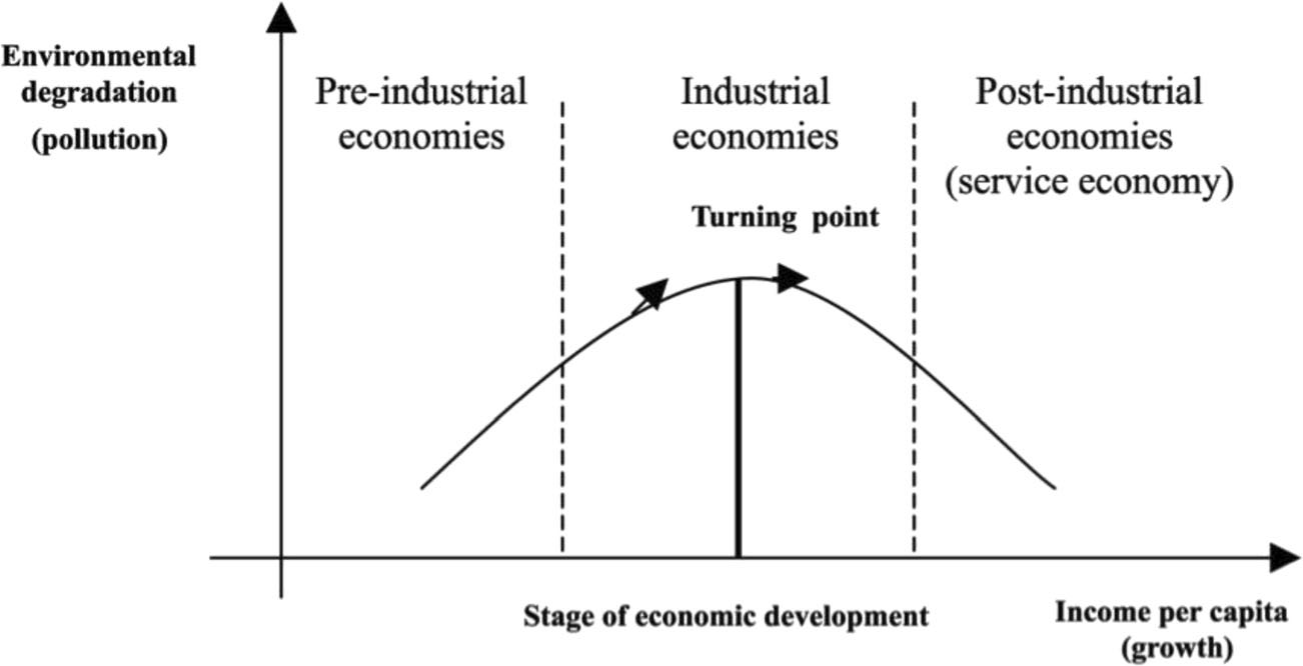
Environmental Kuznets curve. Source: Panayotou (2003)

Because human activities result in environmental degradation through the emission of greenhouse gases, pollution has become a key concept in the paradigm of economic development. In explaining this nexus, the environmental Kuznets curve (EKC) gave an overwhelming insight into the dynamics of interaction between economic growth and environmental pollution. The EKC is credited to Simon Kuznets and later formalized by Grossman and Krueger (1991). The EKC curve shows that human activities that bring about economic growth lead to environmental degradation due to the use of energy inefficient methods in the early stages of productivity. This is relationship is indicated in Fig. 1.

However, it is assumed that the economy advances towards industrialization; there is a tendency for the economy to adopt more energy-efficient methods which bring about a reduction in energy emission. Also, with industrialization, there tends to be a structural transformation where the economy moves from the traditional Agrarian sector to the urbane services sector. At this stage, the services sector is the driver of economic growth.

### Survey of empirical literature

The extant empirical literature on the nexus between energy consumption, economic growth, and greenhouse gas emission produced mixed results. For instance, studies such as Arouri et al. (2012), Salahuddin and Gow (2014), Masih and Masih (1998), Pao and Tsai (2010), Saidi and Hammami (2015), and Apergis and Payne (2010) found evidence that suggests energy consumption have produced positive effect on carbon emissions, lending credence to the argument that energy consumption leads to environmental degradation. This finding is not in tandem with studies such as Acheampong (2018) who found energy consumption as having a negative effect on carbon emissions.

On the relationship between economic activity using GDP as a proxy and carbon emission, Balıbey (2015), Zaman and Moemen (2017), Chaabouni and Saidi (2017), Saidi and Hammami (2015), and Muhammad (2019) all found GDP as having positive effect carbon emission. As the economy grows, carbon emissions also increase. On the other hand, Kasman and Duman (2015) found GDP as having a depressing effect on carbon emissions. Interestingly, studies such as Salahuddin and Gow (2014), Acheampong (2018), Soytas et al. (2007), and Gorus and Aydin (2019) present evidence that shows GDP as having no significant effect on carbon emissions.

In terms of causality, Jahangir Alam et al. (2012) and Halicioglu (2009) found evidence of short run causality running from energy consumption to carbon emissions. Also, the presence of long run causality running in the same direction was found by studies such as Zhang and Chang (2009), Soytas et al. (2007), Menyah and Wolde-Rufael (2010), Omri (2012), and Ssali et al. (2019). In the same vein, studies such as Mirza and Kanwal (2017) and Al-Mulali and Sab (2018) found bidirectional causality between energy consumption and carbon emissions. On causality between GDP and carbon emissions, studies such as Lean and Smyth (2010), Omri (2012), and Al-Mulali and Sab (2018) found evidence of bidirectional causality between GDP and carbon emissions. On the other hand, Soytas et al. (2007) found no evidence of causality between the variables.

Empirical literature that sought to examine the environmental Kuznets curve (EKC) also produced mixed results. Studies such as Narayan and Narayan (2010), Apergis and Payne (2010), Apergis and Ozturk (2015), Balıbey (2015), and Zaman et al. (2016) found the existence of a non-linear relationship between economic growth and carbon emissions, lending credence to the presence of the EKC. However, studies as Abid (2016) did not find evidence that supports the existence of the EKC hypothesis.

Studies that examined the EKC in the context of Africa are also without consensus. For instance, Sulemana et al. (2016) found evidence to support the existence of EKC in African countries. Similarly, Osabuohien et al. (2013) investigated the EKC in a panel of oil-producing and non-oil producing African countries. The findings of their study confirmed the EKC for oil-producing countries; however, no evidence was found for non-oil producing countries. Further, Aiyetan and Olomola (2017) examined the presence or otherwise of the EKC in Nigeria which happens to be an oil-producing country. The result of the study found the presence of an inverted U-shaped relationship between environmental degradation and economic activities, hence confirming the presence of the EKC.

On the other hand, Effiong and Oisaozoje (2016) examine the presence of EKC in a panel of 49 African economies. The result of the study did not find evidence to support the presence of EKC in African economies. In addition, Orubu and Omotor (2011) investigated the EKC in Africa. Although findings of their study indicated the presence of an inverted U-shaped relationship thereby confirming the EKC in the case of suspended particle matter, evidence of EKC was not found in the context of water pollutants. Similarly, Ogundipe et al. (2014) examined the EKC for African economies by building models for 53 African countries and also disaggregate models for low income, lower and upper-middle-income African countries. The result of the study indicated the absence of EKC for the full model of African countries, low income and upper-middle countries; however, evidence of EKC was found for the lower-middle-income countries. Further, Yaduma et al. (2015) investigated the EKC and found no evidence to support the existence of the EKC for African countries. Finally, Adu and Denkyirah (2019) tested the EKC in the context of West African countries and found out that the relationship between economic activities and environmental degradation cannot be explained by an inverted U-shaped curve, hence signifying the absence of the EKC.

## Methodology

### The data

For this study, panel data on the study variables sourced from the World Bank’s World Development Indicator (WDI) database covering the period ranging from 1970 to 2016 was employed.

Existing empirical studies largely rely on the use of CO_2_ as the measure of greenhouse gas emissions. However, this study extends the literature by incorporating more measures of greenhouse gas emissions which include nitrous oxide and methane. The variables included in the study are presented in Table 1.

**Table 1:** Data used

Representation	Variables	Unit of measurement
ECON	Energy consumption	Energy use (kg of oil equivalent per capita)
CO_2_	Carbon dioxide emission	CO2 emissions (metric tons per capita)
N_2_O	Nitrous oxide emission	Nitrous oxide emissions (thousand metric tons of CO2 equivalent)
GDP	Gross domestic product per capita	GDP per capita (constant 2010 US$)
GDPSQ	Squared GDP per capita	GDP per capita squared
METH	Methane emission	Methane emissions (kt of CO2 equivalent)

Sources: World Bank, World Development Indicators. All the variables with the exception methane were converted to their log form

### Model specification and description

This paper extends on the empirical model specification of Apergis and Payne (2009); Apergis and Payne (2010) by taking into consideration other alternative measures of GHG emissions. Similar to Hamit-Haggar (2012). The empirical model of this study is specified as:

1 $$ {\mathrm{GHG}}_{\mathrm{it}}={\upbeta}_i+{\upbeta}_1{\mathrm{ECON}}_{i,t}+{\upbeta}_2{\mathrm{GDP}}_{i,t}+{\upbeta}_3{\mathrm{GDP}\mathrm{SQ}}_{i,t}+{\upvarepsilon}_{\mathrm{it}} $$

where *i*=1,2,……..,*N* and *t*=1,2,…….,*T*

Where GHG_it_ represents greenhouse gases and is disaggregated into carbon dioxide (CO_2_), nitrous oxide (N_2_O), and methane (NH_4_); ECON_it_ denotes energy consumption. GDP_it_ denotes gross domestic product which is used as a measure of economic activity, and GDPSQ_it_ denotes gross domestic product squared which was included to confirm the existence of the EKC, i.e., the presence or otherwise of a turning point in the emission and economic growth relationship.

### Estimation techniques

The inclusion of the lagged dependent variable as one of the explanatory variables makes the model dynamic, and this may result in endogeneity bias, the estimation technique adopted by this study, the panel autoregressive and distributed lag (PARDL) model account for the endogeneity problem. In addition, since the panel is long (with long-time dimension and short cross-sectional components), stationarity and homogeneity of the variables are not certain. Thus, in line with Pesaran et al. (1999); Pesaran and Smith (1995), the study carried out the unit root tests of Rao et al. (2010). This test is constructed as a residual-based Lagrange multiplier test and was applied by this study due to the nature of our panel which is unbalanced; other panel unit root tests such as Levin et al. (2002); Choi (2001); Pesaran (2006) among others all require the panel to be balanced. Having identified our variables to be of mixed order of integration *(I(0)* and *I(1))*, we adopted the panel ARDL proposed by Pesaran et al. (1999), see Abubakar and Shehu (2015). The panel ARDL specification of our model is given as:

2 $$ {\mathrm{ghg}}_{\mathrm{it}}=\sum \limits_{m=1}^p{\phi}_{\mathrm{im}}{\mathrm{ghg}}_{i,t-m}+\sum \limits_{n=0}^q{\lambda}_{\mathrm{in}}{\mathrm{ECON}}_{i,t-n}+\sum \limits_{n=0}^q{\delta}_{\mathrm{in}}{\mathrm{GDP}}_{i,t-n}+\sum \limits_{n=0}^q{\psi}_{\mathrm{in}}{\mathrm{gdpsq}}_{i,t-n}+{\mu}_i+{\varepsilon}_{\mathrm{it}} $$

where*λ*_in_, *δ*_in_,and *ψ*_in_are 1 × *K*vector of coefficients of the regressors, *ϕ*_im_are scalars of the coefficient of lagged dependent variable. Equation 2 is reparametrized to account for both the short run dynamics and adjusting coefficients, the specification is as follows:

3 $$ {\displaystyle \begin{array}{l}\varDelta {\mathrm{ghg}}_{\mathrm{it}}={\alpha}_{1\mathrm{i}}{\mathrm{ghg}}_{i,t-1}+{\alpha}_{2i}{\mathrm{ECON}}_{i,t-1}+{\alpha}_{3i}{\mathrm{GDP}}_{i,t-1}+{\alpha}_{4i}{\mathrm{gdpsq}}_{i,t-1}+\\ {}+\sum \limits_{m=1}^{p-1}{\phi}_{\mathrm{im}}\varDelta {\mathrm{ghg}}_{i,t-m}+\sum \limits_{n=0}^{q-1}{\lambda}_{\mathrm{in}}\varDelta {\mathrm{ECON}}_{i,t-n}+\sum \limits_{n=0}^{q-1}{\delta}_{\mathrm{in}}\varDelta {\mathrm{GDP}}_{i,t-n}+\sum \limits_{n=0}^{q-1}{\psi}_{in}\varDelta {\mathrm{gdpdq}}_{i,t-n}+{\mu}_i+{\varepsilon}_{it}\end{array}} $$

The error correction form is given as;

4 $$ \varDelta gh{g}_{it}={\alpha}_{1i}{\upsilon}_{i,t-1}+\sum \limits_{m=1}^{p-1}{\phi}_{im}\varDelta gh{g}_{i,t-m}+\sum \limits_{n=0}^{q-1}{\lambda}_{\mathrm{in}}{\Delta \mathrm{ECON}}_{i,t-n}+\sum \limits_{n=0}^{q-1}{\delta}_{\mathrm{in}}{\Delta \mathrm{GDP}}_{i,t-n}+\sum \limits_{n=0}^{q-1}{\psi}_{\mathrm{in}}\varDelta {\mathrm{gdpsq}}_{i,t-n}+{\mu}_i+{\varepsilon}_{\mathrm{it}} $$

where *υ*_*i*, *t* − 1_ = ghg_*i*, *t* − 1_ − *ρ*_1*i*_ECON_*i*, *t* − 1_ − *ρ*_2*i*_GDP_*i*, *t* − 1_ − *ρ*_3*i*_gdpsq_*i*, *t* − 1_and long-run coefficients are given as;

$$ {\rho}_{1i}=-\frac{\alpha_{2i}}{\alpha_{1i}},{\rho}_{2i}=-\frac{\alpha_{3i}}{\alpha_{1i}},{\rho}_{3i}=-\frac{\alpha_{4i}}{\alpha_{1i}} $$

## Findings and discussion

### Unit root test result

The study determines the order of integration of the variables by employing the Rao et al. (2010) unit root test with the null hypothesis of series being stationarity. The result is presented in Table 2.

**Table 2:** Unit Root Test Result

	Null hypothesis: no unit root with common unit root process
Variables	Hadri test statistic
Energy consumption	48.1***^a^
CO2	21.87***^b^
N2O	50.39***^b^
GDP	− 0.8281***^b^
GDPSQ	13.7047***^b^
Methane	− 1.4453***^b^

^a, b^Stationarity at level and at first difference respectively

***, **, *Statistical significance at 1%, 5%, and 10% respectively

From the panel, unit root test result presented in Table 2, the null hypothesis of non-stationarity of series is rejected if the computed Hadri statistic is greater than the critical values. The findings of the study show that only energy consumption is stationary at level *(I(0))*, however, the other variables are integrated of order one *(I(1))*, meaning they became stationary after taking their first difference. Since variables are integrated of mixed order, the suitable tool of analysis to apply is the panel ARDL model.

### Panel cointegration test

In an effort to further check for possible cointegration among the variables, the Pedroni panel cointegration test was employed. The three models were subjected to the cointegration, and estimates are presented in Table 3. The estimates also indicate a cointegrating relationship as indicated by the statistical values in Table 3. This is because all the test estimates with the exception of panel t for Co_2_ and nitrous oxide are significant at least at 10% level of significance.

**Table 3:** Panel cointegration test

Test statistics	Panel			Group		
	Co2	Methane	Nitrous	Co2	Methane	Nitrous
v	− 1.078	1.024	− 1.135	–	–	–
Rho	1.466	− 6.41	1.064	2.812	− 3.954	1.963
t	1.599	− 8.708	0.6485	2.671	− 8.095	0.3466
adf	2.697	− 9.165	2.686	2.748	–	1.482

All test statistics are distributed *N* (0,1), under a null of no cointegration, and diverge to negative infinity (save for panel v)

### Hausman test result

The study estimates the models with both the mean group (MG) and pool mean group (PMG) estimators and then subjects them to Hausman test. The Hausman test specifies the null hypothesis of efficient PMG estimators against the alternative hypothesis of a consistent MG estimator. The findings of the Hausman test are presented in Table 4.

**Table 4:** Hausman test result

Variables	Coefficients		Standard errors
	(b) Mean group (MG)	(B) Pooled mean group (PMG)	
ECON	− 0.207	0.009	0.207
GDP	− 0.159	0.012	0.276
GDPSQ	0.014	− 0.0001	0.013
Chi^2^		4.08	
Prob chi^2^		0.253	

*b* Consistent under Ho and Ha; *B* Inconsistent under Ha, efficient under Ho

Table 4 presents the Hausman test result, the test is used to choose between MG and PMG. From the test result, the value of the chi^2^ statistic (4.08) and a corresponding probability value of 0.25 clearly indicates that we do not reject the null hypothesis against the alternative. This signifies the preference of the PMG estimator ahead of the MG estimator, as a result, the focus of the study is on estimates obtained from the PMG estimator. Our discussion of findings will be on these estimates. Also, the Hausman test was estimated in order to choose between the PMG estimator and DFE estimator, the result obtained indicated the choice of PMG over DFE. Although the result presented in Table 4 is that of the Co_2_ model, the Hausman test was estimated for the methane and nitrous oxide models and the results^1^ follows that of Co_2_ model presented above.

### Panel ARDL estimation results

Following findings obtained from the Hausman test, the study presents estimates of both the MG and PMG estimators to aid comparison; however, inferences are based on estimates obtained from the PMG estimator. Short-run and long-run estimates of the three models constituting the components of GHG emissions are presented.

#### Panel ARDL estimation result of carbon dioxide model

Under this model, carbon dioxide is the dependent variable, the impact of energy consumption and economic activity on it was estimated. The result is presented in Table 5.

**Table 5:** Panel ARDL estimates of carbon dioxide model (1, 0, 0, 0) (Selection of optimal lag length is based on the Bayesian Information Criterion (BIC))

	(LR)	(SR)
Variables	PMG	PMG
ECT		− 0.299**
		(0.116)
D (energy consumption)		0.00966
		(0.0122)
D (gross domestic product)		0.000506
		(0.00430)
D (gross domestic product squared)		5.02e–05
		(6.36e–05)
Trend		0.00258
		(0.0197)
Energy consumption	0.00931	
	(0.0138)	
Gross domestic product	0.0116**	
	(0.00528)	
Gross domestic product squared	− 6.92e–05	
	(0.000247)	
Constant		0.482
		(0.918)

Source: Authors’ computation

^***, **, *^Statistical significance at 1%, 5%, and 10% respectively. Standard errors are in parenthesis

Table 5 presents the result of the panel ARDL estimated model. In the long run, PMG estimates of the coefficient of GDP was found to have a significant positive effect on carbon dioxide emissions, showing that an increase in economic activity is associated with rising carbon dioxide emission. This finding is consistent with the result of studies such as Apergis and Payne (2009); Bhattacharyya and Ghoshal (2010); Richmond and Kaufmann (2006). Energy consumption was found to exert an insignificant positive effect on carbon dioxide emissions; this is premised on the positive signed coefficient of the variable. The squared GDP included to examine the EKC has a negative coefficient as theoretically expected, but its statistical insignificance shows we cannot confidently say the EKC-type relationship exists in the case of the selected countries. We could thus infer that in the long run, there is no enough evidence to support the EKC hypothesis in the case of carbon dioxide emissions and that the significant driving force of higher carbon emissions is increase in economic activities.

Estimates from the short-run error correction model show both GDP and energy consumption as producing an insignificant positive effect on carbon emission. The coefficient of squared GDP was also found to be positive and statistically insignificant, further confirming the lack of evidence to support the EKC hypothesis. The error correction term (ECT) which captures the speed of adjustment has the coefficient of − 0.299, this shows that about 30% reversion towards long run equilibrium is completed in a year following a shock to the economy.

#### Panel ARDL result of methane model

As highlighted in the introduction section, one of the significant contributions of this study is the extension of the environmental degradation-economic variables nexus to incorporate other elements of GHG, i.e., methane and N_2_O gas emissions. The results from the estimated panel ARDL methane model are presented in Table 6.

**Table 6:** Panel ARDL estimates of methane model (1, 0, 0, 0) (Selection of the optimal lag length is based on the Bayesian Information Criterion (BIC))

	(LR)	(SR)
Variables	PMG	PMG
ECT		− 0.583***
		(0.205)
D (energy consumption)		1.359
		(0.951)
D (gross dom. product)		0.538**
		(0.267)
D (gross domestic product squared)		− 0.0166
		(0.0113)
Trend		− 0.283**
		(0.134)
Energy consumption	0.0467	
	(0.252)	
Gross domestic product	0.502**	
	(0.205)	
Gross domestic product squared	− 0.0183*	
	(0.0106)	
Constant		8.846**
		(3.852)

Standard errors in parentheses

***p < 0.01, **p < 0.05, *p < 0.1

Table 6 presents the result of the estimated methane model. PMG long run estimates show the coefficient of GDP as having a significant positive effect on methane emissions. This indicates that the higher the economic activity, the higher the methane emission and by extension environmental degradation. This finding is like inferences from the carbon dioxide model. The coefficient of energy consumption, though positive, was found to be statistically insignificant, indicating that energy consumption produces an insignificant positive impact on methane emissions. This finding is like that of the carbon dioxide model. The coefficient of GDP squared was found to be negative and statistically significant, conforming to theoretical expectations of the EKC. This indicates that a turning point exists in the GDP-methane emission relationship, signifying the existence of an inverted U-shaped relationship as postulated by the EKC. We could thus say that using methane as a measure of environmental degradation, there exist evidence to support the EKC hypothesis in the long run.

Estimates of the error correction model show that in the short run, GDP produces a significant positive effect on methane emissions, like findings of the long run model. Similarly, energy consumption, just like in the long run was found to have an insignificant positive impact on methane emissions. The coefficient of squared GDP though negatively signed was found to be statistically insignificant, signifying that there is no evidence to support the EKC hypothesis in the short run. The error correction term has a statistically significant coefficient of − 0.583, indicating that about 58% correction towards long run equilibrium is corrected in a year following a shock to the economy.

#### Panel ARDL result of nitrous oxide model

The other alternative employed was using nitrous oxide emission as a proxy for environmental degradation. The result of the estimated nitrous oxide model is presented in Table 7.

**Table 7:** Panel ARDL estimates of nitrous oxide model (1, 0, 0, 0) (The selection of optimal lag length is based on Bayesian Information Criterion (BIC))

	(LR)	(SR)
Variables	PMG	PMG
ECT		− 0.431***
		(0.166)
D (energy consumption)		− 0.00541
		(0.0139)
D (gross domestic product)		0.0388
		(0.0256)
D (gross domestic product squared)		− 0.00199
		(0.00159)
Trend		− 0.0235
		(0.0508)
Energy consumption	0.0198	
	(0.0612)	
Gross domestic product	0.0383	
	(0.0288)	
Gross domestic product squared	− 0.00403*	
	(0.00243)	
Constant		5.031
		(3.651)

Standard errors in parentheses

***p < 0.01, **p < 0.05, *p < 0.1

Table 7 presents the result of the estimated nitrous oxide model. From the result, PMG long run estimates show GDP as having an insignificant positive effect on nitrous oxide emissions. This finding is not like the results of the carbon dioxide and methane model where GDP had a significant effect. The implication of this finding is that an increase in economic activity does not significantly increase nitrous oxide emissions. In the same vein, energy consumption was found to have an insignificant positive impact on nitrous oxide emissions. GDP-squared coefficient was found to be negative and significant in line with theoretical expectations; however, since the coefficient of GDP was found to be insignificant, there is no enough evidence that supports the EKC hypothesis in the case of nitrous oxide in the long run.

In the short run, GDP was also found to exert an insignificant positive effect on nitrous oxide emission while energy consumption produced an insignificant negative effect on nitrous oxide emissions. The coefficient of GDP squared was found to be negative but insignificant also showing that there is no evidence to support the EKC hypothesis. The error correction term coefficient of − 0.43 showed that about 43% correction towards long run equilibrium is completed in a year following shocks to the economy. The significant positive relationship between these greenhouse gases and economic growth can be attributed to the high rate of economic activities which predominantly involves production using fossil fuels.

## Concluding remarks and policy implications

This study investigated the impact of economic growth and energy consumption on greenhouse gases for OPEC African member countries. The focus on these countries is premised upon the high level of emission of greenhouse gases in them partly since the economies are largely oil-based, with oil exportation taking the largest share of their export components. The study employed various measures of greenhouse gases (that is, CO_2_, ,methane and nitrous oxide) departing from most studies that relied on the use of CO_2_ alone. This provides a wider lens of examining the relationship between economic growth, energy consumption, and environmental degradation.

The findings of the study showed that economic growth which signifies an increase in economic activity impacts negatively on the environment via an increase in carbon emissions. This can be attributed to the fact that the economies in question are oil-based, and production of goods and services are powered via the use of methods that utilizes fossil fuel. Also, gas flaring and other methods of oil extraction contribute to this outcome. The study only found evidence in support of the environmental Kuznets curve hypothesis in the case of methane emissions, no evidence was found in the case of the carbon dioxide and nitrous oxide models. This is an indication that indeed economic activities to a large extent negatively affect the environments, with little evidence of abatement.

From the policy front, this study underscores the need for oil-producing African countries to take positive steps aimed at gradually diversifying their energy sources by investing more in cleaner sources of energy such as biofuel, solar power, and wind energy among others. If implemented, it will go a long way in reducing the amount of fossil fuel utilized for economic activities, thereby reducing greenhouse gas emissions. Also, there is a need for massive investment in abatement technologies aimed at reversing or at least mitigating the negative effect of economic activities on the environment. The long-term implication of this will be the simultaneous realization of economic growth and environmental sustainability, and by extension reversing the ugly trend of climate change.

## Footnotes

1 The results can be provided by the authors upon request.
